# Protective Effects of rLPG3 Plus Freund’s Incomplete Adjuvant on Parasitism, Hepatic Function, and Immune Modulation in Experimental Visceral Leishmaniasis

**DOI:** 10.1021/acsinfecdis.5c01006

**Published:** 2026-01-20

**Authors:** Daniel Silva Sena Bastos, Bianca Meirelles Miranda, Caroline Itagiba Rooke, Neverton José Silva Ferreira, Luiz Otávio Guimarães Ervilha, Renner Philipe Rodrigues Carvalho, Ana Cláudia Ferreira Souza, Mariana Machado Neves, Leandro Licursi de Oliveira, Eduardo de Almeida Marques da Silva

**Affiliations:** † Laboratory of Immunoparasitology, Department of General Biology, Federal University of Viçosa, Viçosa, Minas Gerais, MG, 36570-000, Brazil; ‡ Laboratory of Structural Biology, Department of General Biology, Federal University of Viçosa, Viçosa, Minas Gerais, MG, 36570-000, Brazil; § Laboratory of Structural Biology, Department of Animal Biology, Federal Rural University of Rio de Janeiro, Seropédica, Rio de Janeiro, 23890-000, Brazil; ∥ Laboratory of Immunochemistry and Glycobiology, Department of General Biology, Federal University of Viçosa, Viçosa, Minas Gerais, MG, 36570-000, Brazil

**Keywords:** Visceral leishmaniasis, LPG3, Oxidative Stress, Hepatoprotection, Vaccine

## Abstract

Visceral leishmaniasis (VL) is a neglected tropical disease affecting humans and dogs, particularly in urban settings. Current therapies are limited by toxicity, lengthy regimens, and emerging drug resistance. No human vaccine is available, and only a few licensed formulations exist for canine use. Here, we evaluated a recombinant Leishmania infantum lipophosphoglycan-3 (rLPG3) antigen formulated with Freund’s incomplete adjuvant (FIA) against *Leishmania infantum* challenge in BALB/c mice. The formulation reduced hepatic parasitism, increased antioxidant enzyme activities (superoxide dismutase, catalase, glutathione S-transferase), and raised total antioxidant capacity and hepatic nitrite/nitrate, while lipid and protein oxidation markers remained unchanged. Vaccination preserved liver architecture, lowered AST/ALT, reduced granuloma number and area, and shifted granuloma maturation toward organized lesions with greater macrophage content; PAS staining indicated higher hepatocyte glycogen in the rLPG3+FIA group. Serologically, rLPG3+FIA increased IgG1 and the IgG1/IgG2a ratio, indicating a Th2-skewed profile concomitant with reduced parasitism. Within the constraints of this model, time point, and the proof-of-concept use of FIA, these convergent readouts support rLPG3 as a promising antigen for further preclinical development—prioritizing licensable veterinary adjuvants to enable translation into canine VL vaccines.

## Graphical abstract

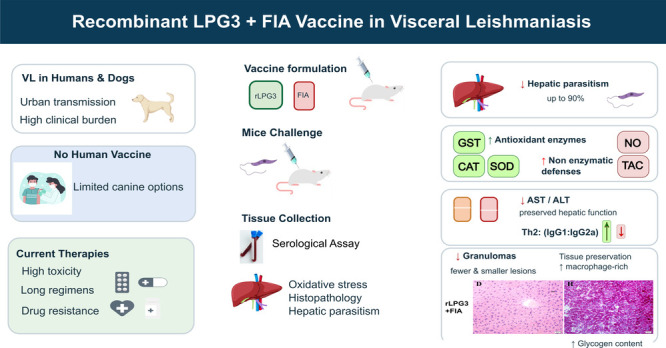

Visceral leishmaniasis (VL), also known as kala-azar, is a severe and often fatal disease caused by protozoan parasites of the genus *Leishmania*, particularly *Leishmania donovani* and *Leishmania infantum* (sin. *L. chagasi*). According to the World Health Organization,^1^ between 50,000 and 90,000 new cases of VL occur each year, with over 95% of these concentrated in countries such as Brazil, India, and Sudan. Despite efforts to control the disease, VL remains endemic in many regions, disproportionately affecting marginalized populations.^2,3^ The disease is characterized by prolonged fever, anemia, hepatosplenomegaly, and, without timely treatment, it leads to high mortality.^1^

Current treatments for VL primarily involve chemotherapeutic agents such as amphotericin B, pentavalent antimonials, and miltefosine.^4^ However, the therapeutic arsenal is limited due to significant drawbacks, including high toxicity, lengthy treatment regimens, and the emergence of drug-resistant *Leishmania* strains.^5–7^ Therefore, there is an urgent need to develop more sustainable solutions to prevent and control VL. Vaccination is a viable strategy, with recent research focusing on subunit vaccines targeting essential parasite proteins, such as lipophosphoglycan 3 (LPG3), a key protein involved in the biology of *Leishmania* parasites.

LPG3 is a conserved protein located internally and on the surface of *Leishmania*, where it plays a crucial role in the entry of the parasite into host cells. Our research group demonstrated that LPG3 contributes to recognizing and entering *Leishmania infantum* into macrophages, a critical step for establishing infection.^8^ Beyond its role in parasite entry, Martins et al.^9^ further characterized the function of LPG3, showing its ATPase activity and ability to bind to heparin, suggesting that LPG3 may also be involved in intracellular processes crucial for parasite survival.

Regarding immunogenic potential, we have explored using native and recombinant LPG3 proteins in vaccine formulations. Emerick et al.^10^ tested the native form of LPG3 combined with adjuvants such as saponin and incomplete Freund’s adjuvant (FIA), demonstrating its ability to induce significant immune responses, including both Th1 and Th17 responses, which are essential for controlling *Leishmania* infections. The recombinant form of LPG3 (rLPG3) has also shown promising results. Bastos et al.^11^ focused on using rLPG3 with saponin as an adjuvant, evaluating not only the immunological effects but also the impact of the vaccine on oxidative stress and liver morphology in murine models of VL. Their findings highlighted the protective effect of this formulation, as it reduced oxidative stress markers and preserved hepatic architecture.

In continuation of prior advancements in LPG3-based vaccine research, this study aims to explore the protective potential of an rLPG3-based vaccine combined with incomplete Freund’s adjuvant in a BALB/c mouse model of visceral leishmaniasis. The primary objectives are to assess the ability of the vaccine to reduce hepatic parasitism, modulate immune responses and protect hepatic function. The results presented in this study show significant reductions in parasitic load, improvements in hepatic function, and evidence of immune modulation, supporting rLPG3 as a promising antigen for the development of subunit vaccines against canine visceral leishmaniasis (CVL).

## Results

### Parasitism and IgG Production

Figure 1 shows data on parasitism, the production of IgG1 and IgG2a antibodies, and the ratio between them in serum samples from *L. infantum-*challenged animals. Vaccinated animals (rLPG3 + FIA) significantly reduced liver parasitism (Figure 1A). On average, compared to the PBS control group, the concentration of tissue parasites decreased by 89% and 95% for the rLPG3 and rLPG3 + FIA groups, respectively. The group immunized with the recombinant protein combined with the adjuvant (rLPG3 + FIA) also showed a marked increase in the production of IgG1 antibodies, if compared to the PBS control (Figure 1B, *p* < 0.05). Therefore, this group also presents a higher IgG1/IgG2a ratio compared to the PBS control group, indicating a shift toward a Th2 immune response (Figure 1C). The other experimental groups, however, displayed antibody levels and ratios that were comparable to those of the control group (*p* > 0.05), suggesting a less pronounced immune modulation.

**Figure 1:**
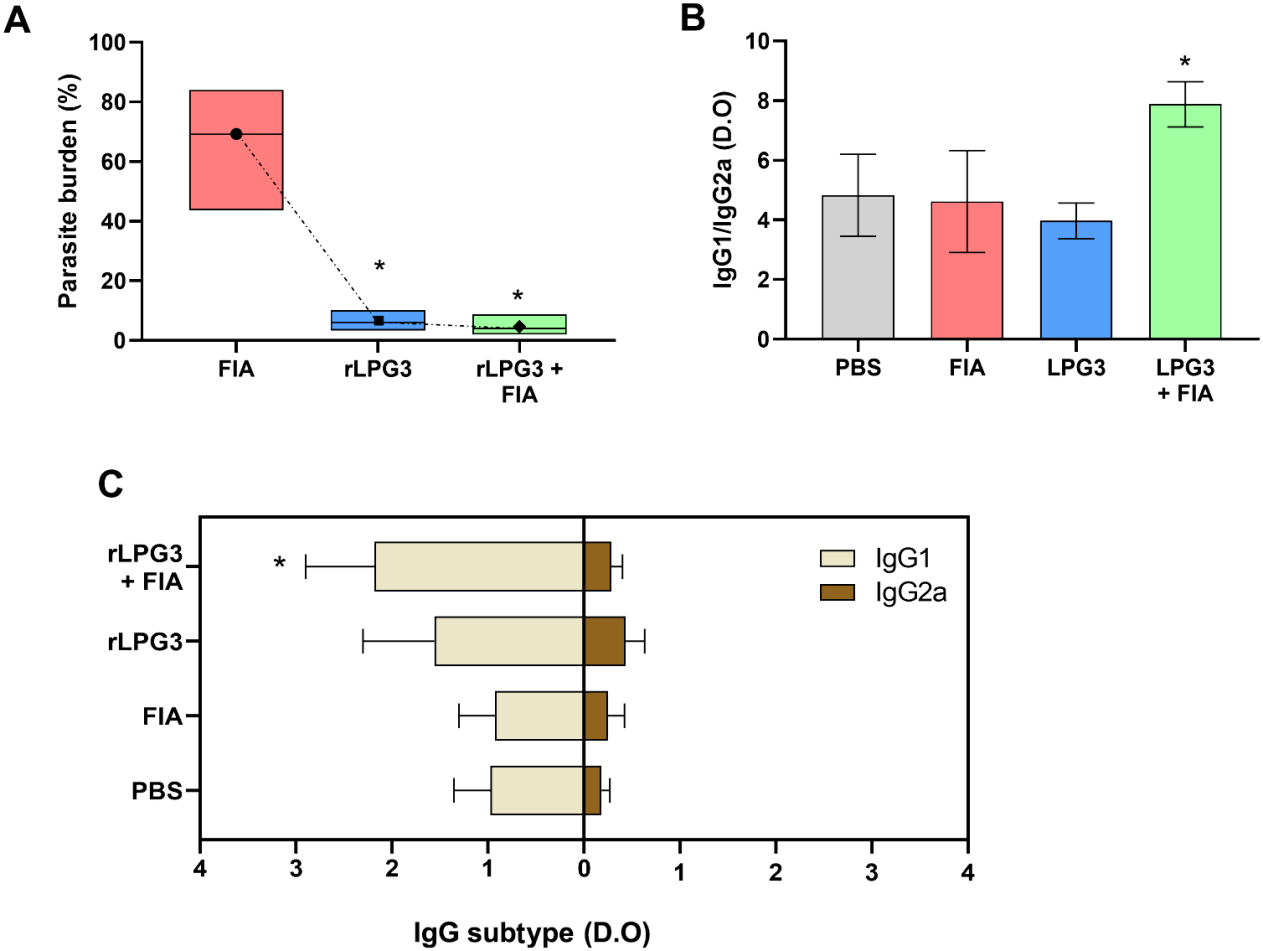
Hepatic parasitism and IgG subclass serum levels in BALB/c mice vaccinated with rLPG3 and challenged with *Leishmania infantum*. (A) Hepatic parasite burden expressed as % of the PBS control. (B) Ratio IgG1/IgG2a. (C) Serum levels of IgG1 and IgG2a. Groups: PBS (vehicle), FIA (incomplete Freund’s adjuvant), rLPG3, rLPG3 + FIA. Data are shown as mean ± SEM; *n* = 6 animals per group. One-way ANOVA followed by Dunnett’s multiple comparisons versus PBS. Asterisks (*) indicate significant differences vs the PBS control (*p* < 0.05).

### Liver Functional Markers

Liver function postchallenge with *L. infantum* promastigote forms was assessed by measuring serum concentrations of AST, ALT, and ALP, which are commonly used as indicators of hepatic health and tissue integrity (Figure 2). FIA, rLPG3, and rLPG3 + FIA exhibited significantly lower AST levels compared to the PBS control group (*p* < 0.05), suggesting reduced liver damage or stress in these groups (Figure 2A). Additionally, the rLPG3 + FIA group also showed a significant reduction in ALT levels, indicating that this vaccine formulation provided superior tissue protection in the animals if compared to the other groups (Figure 2B). Interestingly, ALP and albumin serum levels remained similar across all groups (Figure 2C,D, *p* > 0.05).

**Figure 2:**
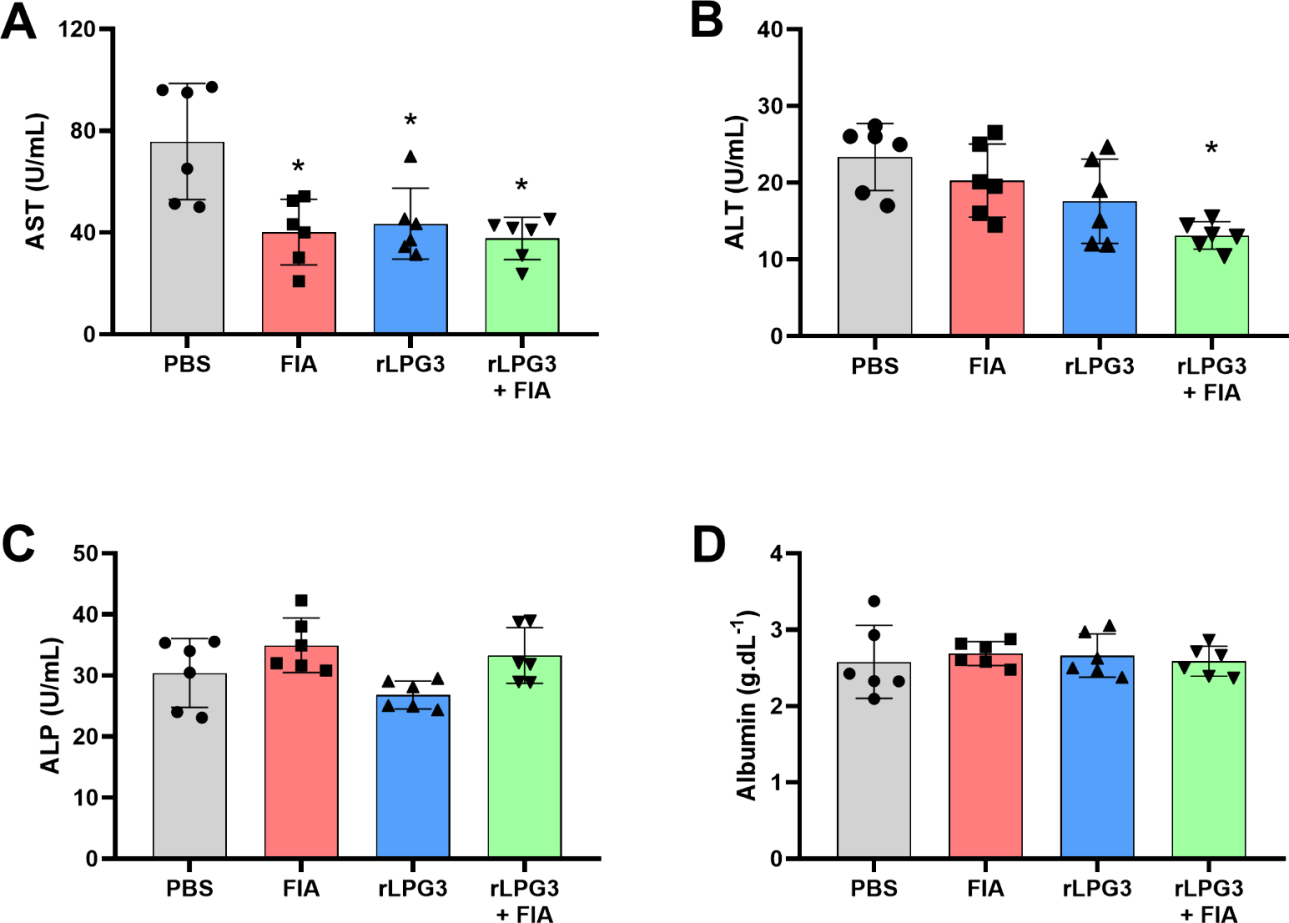
Serum hepatic enzymes in BALB/c mice vaccinated with rLPG3 and challenged with *Leishmania infantum*. (A) AST (aspartate aminotransferase); (B) ALT (alanine aminotransferase); (C) ALP (alkaline phosphatase); (D) Albumin. Data are shown as mean ± SEM; *n* = 6 per group. One-way ANOVA + Dunnett vs PBS. Asterisks (*) indicate significant differences vs the PBS control (*p* < 0.05).

### Oxidative Status

The redox balance was evaluated by measuring the activity of key antioxidant enzymes and markers of oxidative and nitrosative stress, as shown in Figures 3 and 4. Animals vaccinated with rLPG3 + FIA exhibited, after challenge with *L. infantum* promastigote forms, in comparison to the control group, a significant increase in the amount of superoxide dismutase (SOD) and catalase (CAT), enzymes that play a critical role in neutralizing reactive oxygen species (ROS) and preventing oxidative damage to cells (Figure 3A and B, *p* < 0.05). The FIA group presented the same profile to the SOD evaluation (Figure 3A). Additionally, glutathione S-transferase (GST) production was also markedly elevated across all treated groups, except FIA, compared to the control (Figure 3C, *p* < 0.05).

**Figure 3:**
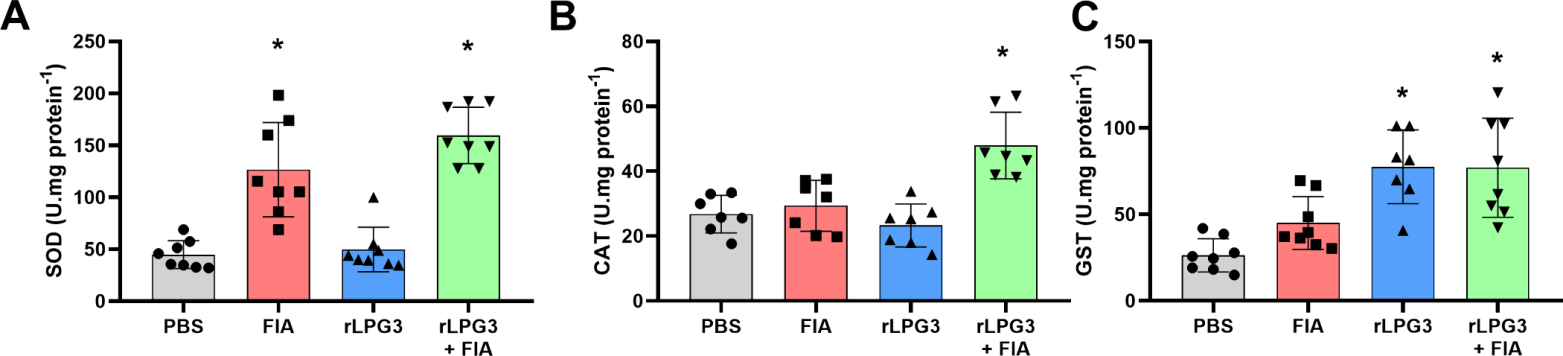
Antioxidant enzyme activities in liver tissue of BALB/c mice vaccinated with rLPG3 and challenged with *Leishmania infantum*. (A) SOD (superoxide dismutase); (B) CAT (catalase); (C) GST (glutathione S-transferase). Data are shown as mean ± SEM; *n* = 8 per group. One-way ANOVA + Dunnett vs PBS. Asterisks (*) indicate significant differences vs the PBS control (*p* < 0.05).

**Figure 4:**
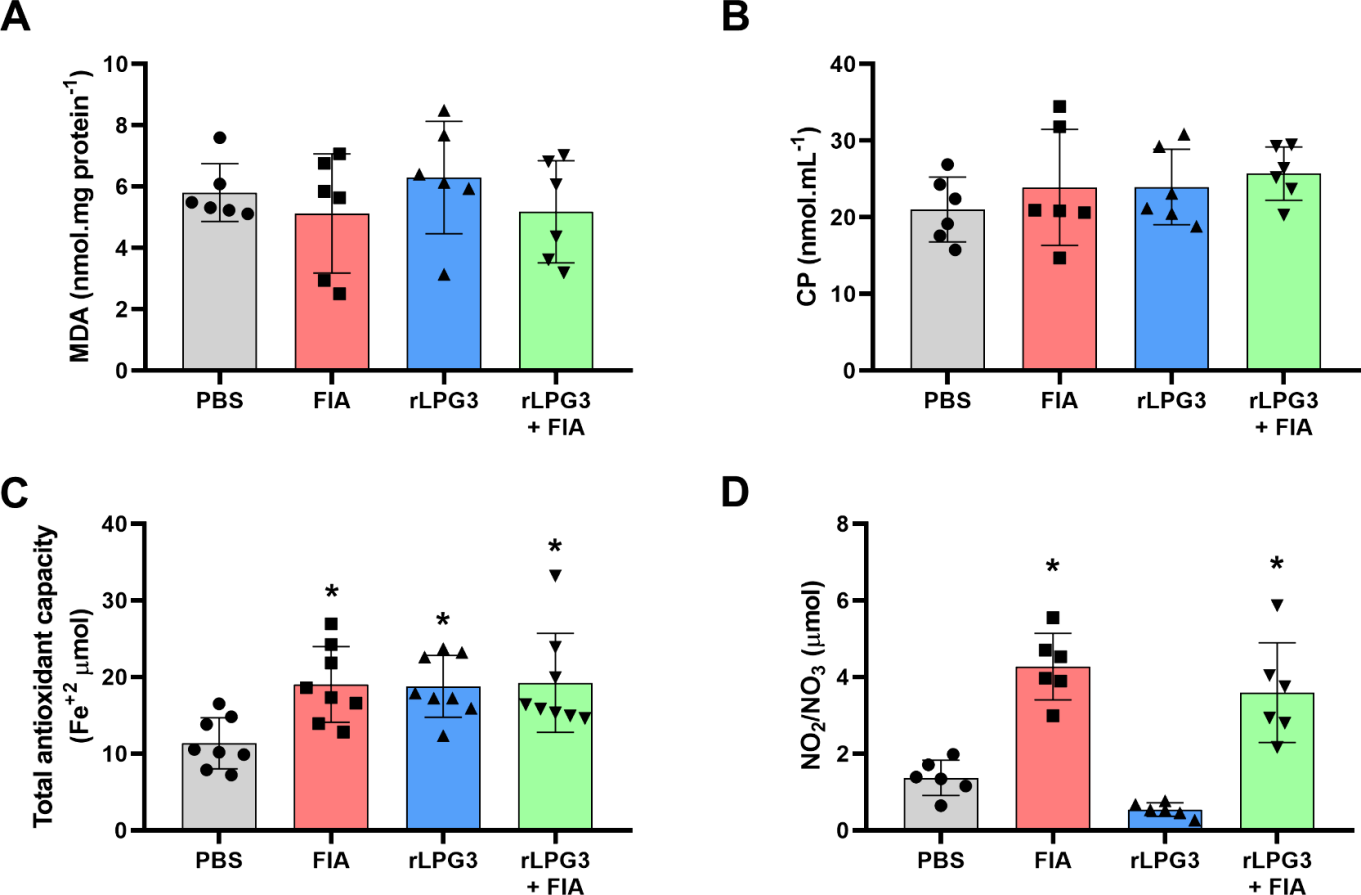
Oxidative and nitrosative stress markers in liver tissue of BALB/c mice vaccinated with rLPG3 and challenged with *Leishmania infantum*. (A) MDA (malondialdehyde); (B) CP (protein carbonyls); (C) Total antioxidant capacity; (D) NO_2_/NO_3_ (nitrite + nitrate). Data are shown as mean ± SEM; *n* = 8 per group. One-way ANOVA + Dunnett vs PBS. Asterisks (*) indicate significant differences vs the PBS control (*p* < 0.05).

Markers of lipid peroxidation (indicative of membrane damage) and protein carbonylation (a marker of oxidative protein damage) showed no significant differences between the groups (*p* > 0.05), suggesting that lipid and protein oxidative damage remained controlled across all experimental conditions (Figure 4A-B). Notably, both the FIA and rLPG3 + FIA groups exhibited an increase in NO levels (*p* < 0.05), which may reflect enhanced immune responses or endothelial activity (Figure 4D). Interestingly, the total antioxidant capacity was increased in all immunized groups compared to the PBS control group (Figure 4C, *p* < 0.05).

### Granuloma Analysis

Absolute number of hepatic granulomas (10 random fields per animal; Figure 5A), the cross-sectional area of individual lesions (≥20 granulomas per group Figure 5B), and their maturation stage (immature vs mature; Figure 5C) were quantified. Compared with PBS (7.50 ± 1.87 granulomas; 3375 ± 2066 μm^2^; 20% mature/80% immature), the rLPG3 + FIA group exhibited fewer granulomas (1.33 ± 0.52; *p* < 0.05), smaller lesions (1301 ± 798.7 μm^2^; *p* < 0.05), and a clear shift toward maturation (70% mature/30% immature). Animals immunized with rLPG3 alone also showed fewer and smaller granulomas relative to PBS (2.17 ± 0.75; 1212 ± 1145 μm^2^; both *p* < 0.05), with a more balanced maturation profile (55% mature/45% immature). In contrast, the FIA group did not differ from PBS in granuloma number or area (5.83 ± 1.47; 2478 ± 2115 μm^2^; *p* > 0.05) and displayed intermediate maturation proportions (35% mature/65% immature).

**Figure 5:**
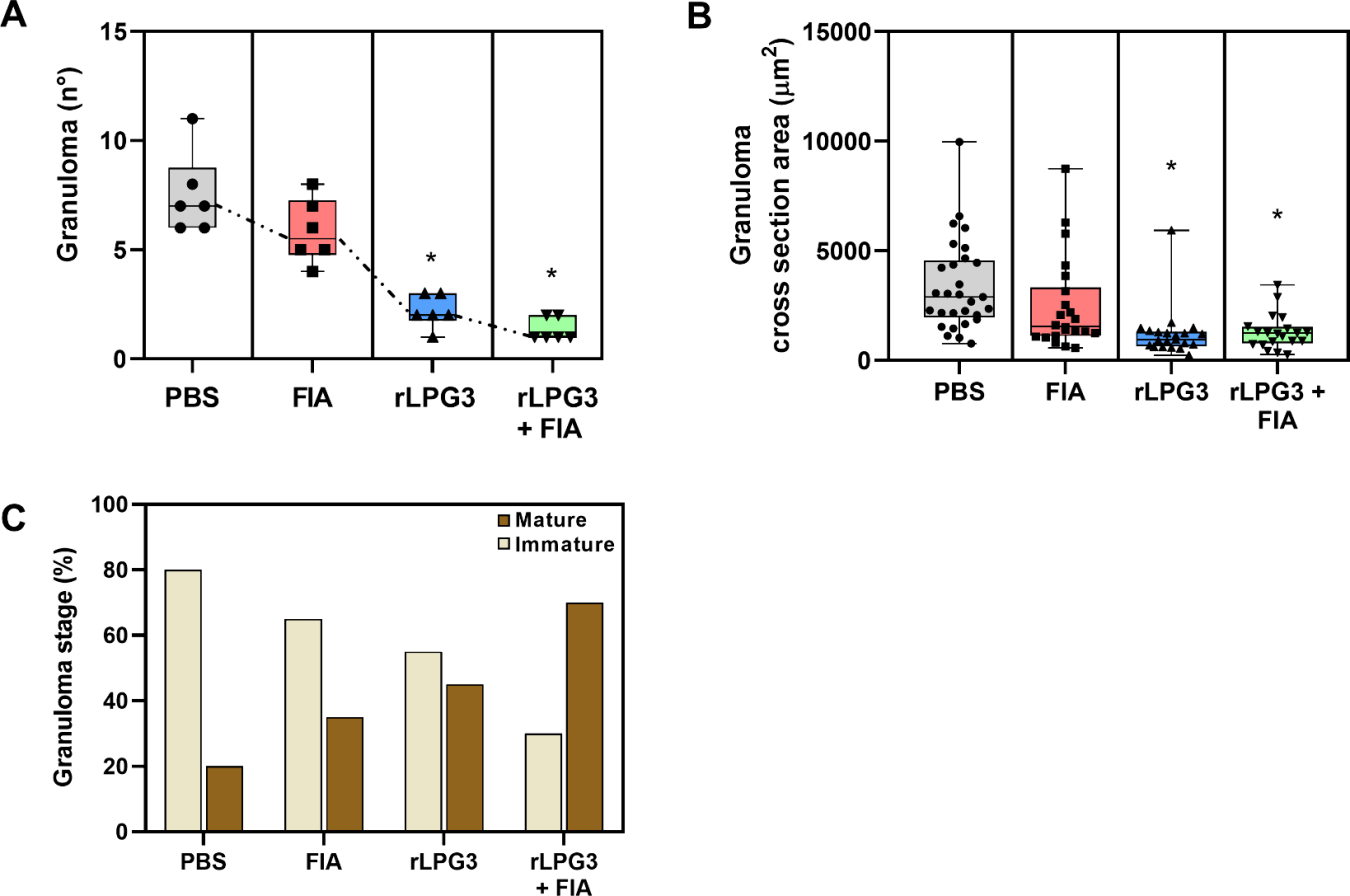
Quantification of hepatic granulomas. (A) Absolute number of granulomas per animal, based on counts from 10 random fields. (B) Cross-sectional area (μm^2^) of individual granulomas (≥20 lesions per group). (C) Granuloma stage categories (immature and mature); percentages calculated from at least 20 granulomas per group. PBS: nonvaccinated (PBS-treated); FIA: treated with Freund’s Incomplete Adjuvant; rLPG3: immunized with rLPG3; rLPG3 + FIA: immunized with rLPG3 plus FIA. * indicates statistical difference (*p* < 0.05) compared to control, determined by ANOVA followed by Dunnett’s multiple comparisons test.

### Stereological Parameters

Histological examination revealed marked hepatic tissue remodeling in animals vaccinated with the recombinant protein plus adjuvant (Figures 6 and 7). In the rLPG3 + FIA group (Figure 6A), stereological analysis showed reduced inflammatory infiltrates (0.272 ± 0.192%), increased sinusoidal capillary proportion (21.40 ± 0.885%), and higher macrophage content (2.11 ± 0.538%), compared to the PBS group. In contrast, PBS-treated animals exhibited greater inflammatory infiltrates (2.998 ± 1.296%), lower macrophage content (1.062 ± 0.183%), and a smaller sinusoidal capillary proportion (17.81 ± 1.737%). Notably, the rLPG3 + FIA group (Figures 6B and 7H) displayed a significantly more intense PAS signal (15.16 ± 1.01%), indicating higher glycogen accumulation compared to the PBS group (8.70 ± 1.95%).The rLPG3 group (Figures 6A and 7C) demonstrated a trend toward tissue normalization, with reductions in the volumetric proportion of inflammatory infiltrates (0.99 ± 0.70%) and of sinusoidal capillaries (15.63 ± 1.390%) relative to PBS (2.998 ± 1.296% and 17.81 ± 1.737%, respectively). PAS staining (Figures 6B and 7G) intensity in this group (9.41 ± 1.66%) was comparable to PBS (8.70 ± 1.95%).

**Figure 6:**
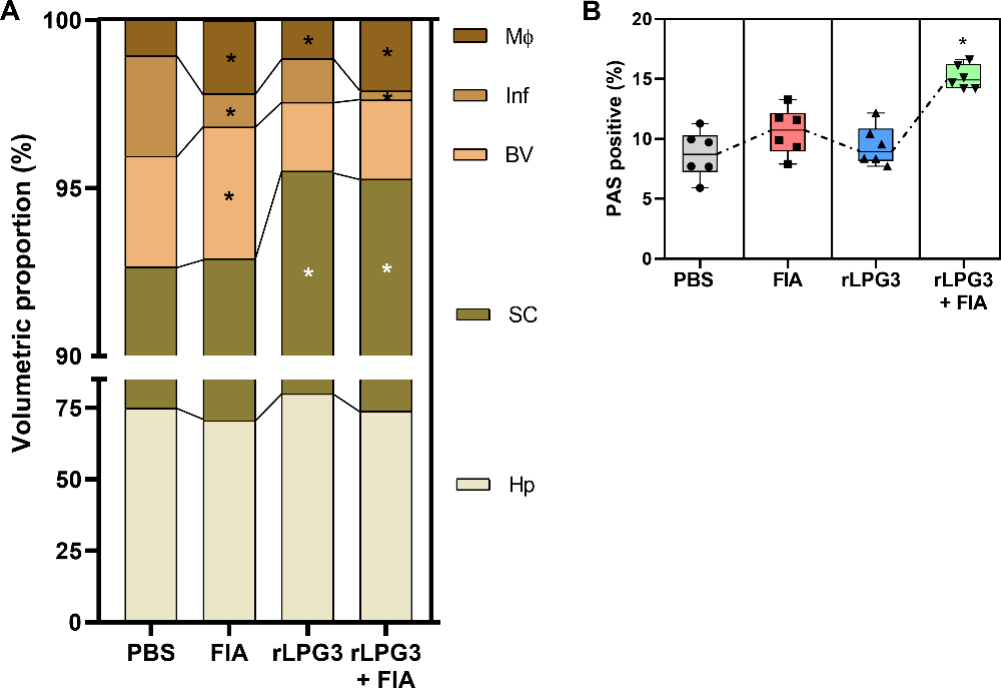
Stereological and quantitative histology of the liver components in BALB/c mice vaccinated with rLPG3 and challenged with *Leishmania infantum*. (A) Volumetric proportions of liver components: Hp (hepatocytes), SC (sinusoidal capillaries), BV (blood vessels), Inf (inflammatory infiltrate), MΦ (macrophages). (B) Glycogen content (PAS-positive area). Data are presented as box-and-whisker plots (box = 25th–75th percentiles; center line = median; whiskers = minimum to maximum), with all individual data points displayed; animals per group; *n* = 6 per group. One-way ANOVA + Dunnett vs PBS. Asterisks (*) indicate significant differences vs the PBS control (*p* < 0.05). Quantitative descriptors correspond to the representative images in Figure 6.

**Figure 7:**
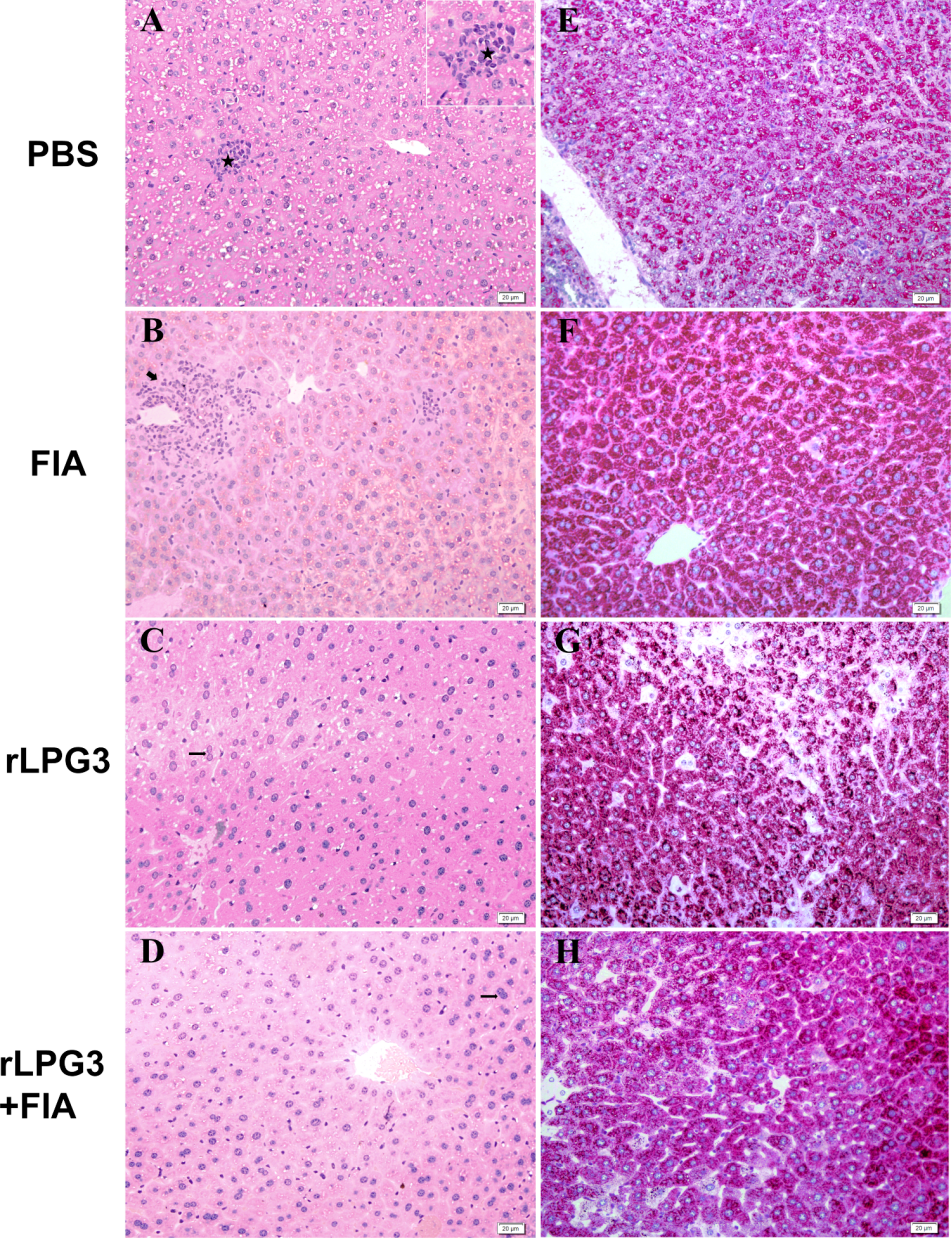
Representative liver photomicrographs (HE and PAS) from BALB/c mice vaccinated with rLPG3 and challenged with *Leishmania infantum*. Panels A–D: HE staining; Panels E–H: PAS staining. Symbols: ★ granulomas; thick arrow, inflammatory infiltrate; thin arrow, binucleated hepatocyte; inset, granuloma detail. Scale bars = 50 μm. Quantification and statistical significance are reported in Figure 5.

Interestingly, animals treated with adjuvant alone (FIA) also exhibited a decrease in inflammatory infiltrates (1.00 ± 0.62%), along with increased blood vessel area (2.05 ± 0.88%) and macrophage content (1.14 ± 0.20%), suggesting a role for the adjuvant in modulating the immune microenvironment and promoting vascularization (Figure 6A). PAS staining intensity in the FIA group (10.62 ± 1.94%) was likewise similar to that of the PBS group (Figures 6B and 7).

## Discussion

The data from this study underscore the effectiveness of the rLPG3 + FIA vaccine formulation in modulating immune response and providing significant hepatic protection against *Leishmania infantum* infection in mice. One of the primary outcomes was the marked reduction in hepatic parasitism and granuloma formation observed in vaccinated groups that received rLPG3 alone or in combination with FIA, highlighting the robust protective potential of these formulations.

The parasite burden in this study was estimated using the LDA, a classical method that quantifies viable parasites but is known to have lower sensitivity and precision at low parasite densities compared with molecular approaches.^12–14^ We acknowledge that LDA can underestimate parasite load in low-burden tissues and is time-consuming, culture-dependent, and prone to higher inter-replicate variability. By contrast, tissue qPCR consistently shows higher analytical sensitivity, wider dynamic range, and superior performance for detecting low parasite densities in experimental leishmaniasis.^15,16^ Notwithstanding these limitations, hepatic parasite burden was consistently lower in mice immunized with rLPG3 than in adjuvant-only and PBS controls, a difference readily detected by LDA and concordant with the histological and biochemical readouts reported in this study.

Recent vaccine studies in experimental VL explicitly pair qPCR-based parasite quantification with macrophage-centered readouts. For instance, Devender et al.^17^ measured hepatic parasite burden by kDNA qPCR and, in the same model, reported reduced granuloma counts and higher NO/ROS production after immunization with the tuzin protein, indicating enhanced macrophage effector functions. Similarly, a chimeric multiepitope protein (rCHI) reduced organ parasitism when parasite load was quantified by qPCR alongside limiting dilution.^18^ Complementarily, Pandey et al.^19^ evaluated vaccine-driven changes on CD14^+^ monocyte/macrophage activation markers and the parasitic load in macrophages, supporting the link between vaccination, macrophage functionality, and parasite control. Earlier A2-based vaccine work also leveraged qPCR to document significant reductions in splenic parasite burden in vaccinated mice.^20^

The translational context is CVL, given the role of dogs as the main domestic reservoir of *Leishmania infantum*.^21^ In this mouse model, rLPG3 formulated with FIA was associated with lower hepatic parasitism and fewer granulomas, alongside lower AST/ALT and higher SOD, CAT, and GST activities. These outcomes support antigen-linked protection within the limits of this data set and the use of a research adjuvant. Prior murine work with rLPG3 plus saponin similarly reduced parasitism and modulated redox markers.^10,11^ Across these data sets, rLPG3 shows repeatable biological activity under distinct adjuvant contexts. Considering CVL as the intended application, evaluating rLPG3 with licensable veterinary adjuvants in canine formulations is a reasonable next step, but it was not addressed in the present study. In mice, the class-switching of antibodies to IgG1 and IgG2a is due to the action of Th2 and Th1 cytokines, respectively, on B lymphocytes. The dosage of these two subclasses of antibodies, therefore, allows us to infer which type of Th1 or Th2 cellular response predominates, for example, in mice subjected to stimulation by a vaccine.^22,23^ Our results demonstrated a significant increase in IgG1 production in the group vaccinated with rLPG3 + FIA, along with unchanged IgG2a levels compared to the other groups, indicating a Th2-directed immune response induced by the vaccine. This shift toward a Th2 profile is associated with antibody-mediated immunity, contrasting with the traditionally protective Th1 response, known for resolving intracellular parasitic infections through macrophage activation and the production of IFN-γ.^24^ Although FIA is effective at inducing strong humoral responses, such as IgG1 production, this may not be advantageous for intracellular infections like leishmaniasis, where robust cellular immune responses are critical.^25^

Considering the evidence from studies on CVL, the correlation between IgG subclasses and disease progression further underscores the complexity of immune responses.^26^ In canine visceral leishmaniasis, subclass associations vary across studies. Several reports link IgG1 to clinical susceptibility, whereas IgG2 is often observed in protective or vaccine-induced responses; nonetheless, reviews note contradictory patterns depending on antigen, assay, and disease stage.^27^

In a vaccine experiment using peptides derived from the Cysteine Protease A protein (CPA160–189) of *Leishmania infantum* in a murine model, a humoral response pattern characterized by high levels of IgG1 was observed in the group immunized with the peptide in association with the adjuvant, without a significant increase in IgG2a levels.^24^ Despite this humoral response profile, a significant reduction in parasitism was observed in the immunized animals compared to the control groups.^28^ This apparent dichotomy reflects the complexity observed in our results, where elevated IgG1 levels and a Th2-directed response were associated with effective parasite control. Therefore, our data aligns with some hypotheses suggesting that both IgG1 and IgG2 subclasses may play roles in immune protection, possibly through distinct but complementary mechanisms.^29–31^

Independent of the IgG subclass profile or immune polarization, vaccinated animals, most notably the rLPG3 + FIA, group exhibited lower serum AST and ALT than adjuvant-only and PBS controls at the sampling time point, indicating reduced hepatocellular injury. These enzymes are key biomarkers, and their elevated levels typically reflect liver damage, often resulting from parasitic infection such as *Leishmania infantum*.^28^ The significant decrease in AST and ALT levels in the rLPG3 + FIA group suggests that the vaccine not only controls parasitic load but also prevents the extensive liver damage commonly observed in nonimmunized infected animals. This reduction in liver enzyme markers aligns with the histopathological findings of reduced inflammatory infiltrates and enhanced tissue recovery, further supporting the protective effect of the vaccine on hepatic integrity.

In contrast, alkaline phosphatase (ALP) and albumin levels did not show significant changes across the experimental groups. ALP is primarily associated with bile duct function,^29^ and its stability across groups suggests that the vaccination did not affect biliary health. Similarly, albumin, a marker of liver synthetic capacity,^32^ remained unchanged, indicating that the protective effects of the vaccine were more localized to controlling cellular damage and inflammation rather than impacting overall liver metabolism or protein synthesis. It is worth noting that, although not measured in the present study, Bastos et al.^11^ observed similar ALP levels in the LPG3 plus saponin vaccinated group compared to their noninfected counterparts. Consistent with these biochemical signals, hepatic redox readouts showed context-dependent modulation.

Vaccination increased hepatic SOD, CAT and GST activities, whereas MDA and protein carbonyls were unchanged across groups. We acknowledge this apparent discrepancy and interpret it as an adaptive antioxidant response at the 28-dpi sampling that limits the propagation of reactive species to stable end-products—a pattern widely described in liver redox biology.^33^ Importantly, unchanged MDA/carbonyls do not preclude oxidative stress: TBARS/MDA and DNPH-carbonyl assays have modest specificity and integrate damage over time, and a single terminal time-point may miss transient or compartment-restricted events.^34,35^ When present, lipid or nitrative damage is more sensitively captured by F2-isoprostanes and 4-HNE-protein adducts, and by 3-nitrotyrosine, respectively.^36^ Thus, the observed enzyme upregulation is compatible with effective redox containment rather than greater oxidative injury at this time-point, although kinetics and causality will require time-course analyses with these specific markers

In terms of specific oxidative and nitrosative stress markers, nitric oxide (NO) production was significantly elevated in both the FIA and rLPG3 + FIA groups. NO is a reactive nitrogen species (RNS) that participates in host defense, particularly against intracellular pathogens such as *Leishmania*.^37,38^ The increased NO levels in the vaccinated groups likely contributed to the observed reduction in parasitism, as NO can directly inhibit *Leishmania* replication within infected cells by inducing oxidative stress in the parasite.^39^ The fact that NO production was also heightened in the FIA-only group suggests that the adjuvant independently enhances innate immune responses, possibly by activating macrophages. However, the combination of rLPG3 and FIA appears to create a synergistic effect that boosts parasite clearance and protects against infection. This modulation of NO, alongside the antioxidant enzyme activity, illustrates the ability of the vaccine to maintain redox balance, limit oxidative damage, and enhance the immune response of the host, resulting in more effective parasite control.

The increased total antioxidant capacity observed in the vaccinated groups, particularly rLPG3 and rLPG3 + FIA, highlights the protective effects of immunization against oxidative stress associated with *Leishmania* infection. Unlike the control group, which demonstrated reduced antioxidant capacity due to heightened oxidative stress and uncontrolled infection, vaccinated animals exhibited a preserved redox balance. This improvement aligns with the observed reduction in granuloma formation, a hallmark of chronic inflammation.^40,41^ Granulomas, formed in response to persistent parasitism, drive intense oxidative activity^42^ and the consumption of antioxidants. By reducing granuloma formation, rLPG3 + FIA vaccination minimizes the consumption of antioxidants, such as glutathione and vitamins, preserving the capacity of the host to counteract oxidative stress while maintaining effective immune defense.

Histological analysis corroborates the findings above and clarifies the tissue-level context of protection. Vaccinated groups—particularly rLPG3 + FIA—displayed fewer hepatic granulomas, reduced granuloma size, lower parasite burden, and greater macrophage content, indicating granuloma remodeling consistent with parasite control.^40,41^ Importantly, granuloma maturation also shifted: rLPG3 + FIA showed a predominance of mature lesions (≈70% mature/30% immature) versus PBS (≈20%/80%). In experimental visceral leishmaniasis, mature granulomas are the organized sites where macrophages more effectively restrict parasite growth, and macrophage-derived nitric oxide contributes to microbicidal activity while constraining collateral tissue damage.^43–46^ In our data set, higher hepatic nitrite/nitrate (NOx) coincided with reduced parasitism, fewer/smaller granulomas, and a greater proportion of mature lesions, supporting the interpretation that vaccination promoted more organized, macrophage-competent granulomas.

Liver macrophages—resident Kupffer cells and recruited monocyte-derived macrophages—organize the formation and maturation of hepatic granulomas.^47^ Pan-macrophage markers such as F4/80 or CD68 report on macrophage content in liver tissue.^48^ By contrast, iNOS (NOS2) and arginase-1 (Arg1) inform microbicidal versus tissue-repair/permissive programs in leishmaniasis.^49,50^ In experimental VL, immature granulomas enriched in Kupffer cells and a transient iNOS peak precede collagenized ‘clear’ granulomas,^40^ whereas Arg1/STAT6-driven pathways have been implicated in niches of parasite persistence.^50^ Although phenotyping was not feasible here, our stereology-based increase in macrophage content, the reduction in granuloma number/size, and the NOx profile are consistent with a shift toward microbicidal macrophage activity within granulomas. A focused IHC/IF panel (F4/80 or CD68 with iNOS and Arg1) would directly test this inference and link granuloma remodeling to intragranulomatous macrophage programs. Furthermore, the vaccinated groups exhibited increased hepatic glycogen content, as visualized by PAS staining. Hepatocyte glycogen varies with metabolic demands and inflammatory signaling, and increased PAS reactivity may reflect altered carbohydrate metabolism during infection resolution. In our context, the concomitant reduction in parasite burden, lower AST/ALT, and attenuated inflammatory infiltrates suggest that vaccination mitigated hepatic stress, which could secondarily favor glycogen replenishment; however, mechanistic confirmation would require targeted metabolic assays.^51^ This finding complements the antioxidant data, reinforcing the idea that vaccination alleviates both oxidative and metabolic stress without impairing the protective response of the host.

These histological and biochemical findings are consistent with previous research on LPG3-based vaccines,^10,11^ which similarly demonstrated improved tissue architecture and immune modulation. Together, they highlight the dual action of rLPG3 and rLPG3 + FIA vaccines in enhancing parasite clearance while protecting host tissues. This comprehensive protection underscores the potential of the rLPG3 vaccine as a candidate for CVL immunization strategies, offering a balanced approach to reducing parasitic burden and preserving host homeostasis.

## Conclusion

In a BALB/c model of visceral leishmaniasis caused by *Leishmania infantum*, immunization with rLPG3—most notably when formulated with Freund’s incomplete adjuvant—was associated with lower hepatic parasite burden by limiting dilution, fewer and smaller granulomas, improved biochemical indicators of hepatocellular injury (reduced AST and ALT), increased activities of SOD, CAT, and GST, higher NOx and total antioxidant capacity, and preserved tissue architecture with greater macrophage content at 28 dpi. Taken together these convergent readouts are consistent with antigen-linked protection and tissue preservation.

The antibody profile in rLPG3 + FIA vaccinated mice was Th2-skewed (increased IgG1 without detectable IgG2a change), yet protection was observed, indicating that effective control may arise from complementary humoral and macrophage-linked mechanisms. FIA is a research-only adjuvant, and used as proof-of-concept for rLPG3 as a candidate antigen with translational relevance to CVL. Next steps include advancing rLPG3 with licensable veterinary adjuvants in canine formulations, incorporating tissue qPCR alongside limiting dilution to extend sensitivity at low parasite burdens, performing time-course studies of redox and damage markers, phenotyping hepatic macrophages (e.g., F4/80/CD68 with iNOS/Arg1) to mechanistically link granuloma remodeling to effector programs. Collectively, the present findings warrant further preclinical development of rLPG3 with compatible formulations.

## Methods

### Animals and Ethics Statement

Female BALB/c mice, aged between 5 to 8 weeks, were sourced from the Central Animal Laboratory at the Center of Biosciences and Health, Federal University of Viçosa, Brazil. The mice were housed under standard conditions of temperature control (25 ± 2 °C) and maintained on a 12-h light/dark cycle. Food and water were provided *ad libitum*. All animal procedures followed ethical principles according to the Veterinary Professional Code of Conduct and were approved by the Ethics Committee on Animal Use (CEUA/UFV, protocol number 16/2016). The study adhered to Brazilian Law Number 11.794 (October 08, 2008), guidelines from CONCEA/MCTI, and the “Diretriz Brasileira de Prática para o Cuidado e a Utilização de Animais para Fins Científicos e Didáticos” (DBCA), including CONCEA’s recommendations for euthanasia protocols.

### Parasite

Promastigotes of *Leishmania infantum* (strain M2682, MHOM/BR/75/M2682) were cultivated in Grace’s insect medium (GIBCO BRL, Grand Island, NY, USA) enriched with 10% heat-inactivated fetal calf serum (FCS; LGC Biotecnologia, Cotia, SP, Brazil), 2 mmol × L^–1^ l-glutamine (GIBCO BRL), and 100 U × mL^–1^ penicillin G potassium (USB Corporation, Cleveland, OH, USA), pH 6.5. Cultures were incubated at 26 °C. Parasite infectivity was maintained via passages in mice.

### Experimental Design

The immunization protocol involved three doses administered biweekly. On day 42 (approximately 2 weeks postfinal dose), the mice were challenged intravenously with 1 × 10^7^ *L. infantum* promastigotes via the lateral tail vein. Mice were assigned to four experimental groups (n = 4 per group) as follows: nonvaccinated (NV; treated with PBS); FIA (50 μg of Incomplete Freund’s adjuvant per dose, administered subcutaneously); rLPG3 (40 μg of rLPG3 administered intraperitoneally in the first dose, followed by two boosters of 20 μg); and FIA + rLPG3 (vaccinated with rLPG3 in the same doses as previously mentioned, combined with Incomplete Freund’s adjuvant). All experiments were conducted in duplicate.

### Production of rLPG3

The recombinant LPG3 (rLPG3) protein was produced as previously described by Martins et al.^9^ In brief, the LPG3 gene was synthesized and inserted into the pUC19 vector, followed by subcloning into the pET 28a^+^ vector. This construct was propagated in *Escherichia coli* DH5α cells (GenOne, Rio de Janeiro, RJ, Brazil). Expression of the recombinant protein was carried out using *E. coli* Rosetta (DE3) cells transformed with the pET 28 plasmid. Following sonication to lyse the cells, the soluble protein fraction was isolated and purified using affinity chromatography with Ni-NTA, heparin-agarose, and a Superdex 200 column. Purity was confirmed via SDS-PAGE with Coomassie staining.

### Euthanasia and Tissue Collection

Mice were euthanized 28 days postchallenge via cervical dislocation. Blood samples were obtained by cardiac puncture and centrifuged at 3000 × *g* for 10 min to separate serum, which was stored at 4–8 °C for subsequent analysis of hepatic function markers and antibody quantification. Livers were excised, sectioned, and weighed. One portion was frozen in liquid nitrogen and stored at – 80 °C for enzymatic assays. Another portion was fixed in Karnovsky’s solution (2.5% glutaraldehyde, 4% paraformaldehyde in 0.1 mmol × L^–1^ phosphate buffer, pH 7.2) for histopathological analysis. A third fragment was used for parasite load assessment in Grace’s insect medium, as described below.

### Parasite Load Determination

Liver parasitism was quantified by limiting dilution assay (LDA) as outlined by Marques-da-Silva et al.^52^ Briefly, liver fragments were homogenized and resuspended in 500 μL of Grace’s insect medium supplemented with 10% FCS, 2 mmol/ × L^–1^ l-glutamine, and 100 U × mL^–1^ penicillin G potassium. Serial 5-fold dilutions were plated in 48-well plates and incubated at 25 °C for 2 weeks. Parasite growth was microscopically observed, and the highest dilution showing promastigote presence was used to estimate the number of parasites per milligram of liver tissue.

### Serological Assay for Antibody Detection

Antibodies specific to rLPG3 were measured using enzyme-linked immunosorbent assay (ELISA), following the method described by Voller et al.^53^ Microplates were coated overnight at 4 °C with 1.0 μg × mL^–1^ of rLPG3 antigen. After blocking with PBS containing 1% gelatin, diluted serum (1:40) was added to the wells, followed by incubation with peroxidase-conjugated goat antimouse IgG1 or IgG2a antibodies. The reaction was developed using OPD substrate, stopped using H_2_SO_4_, and the optical density was measured at 490 nm.

### Hepatic Biomarkers

Serum samples were analyzed for aspartate aminotransferase (AST), alanine aminotransferase (ALT), and alkaline phosphatase (ALP) levels, using commercial biochemical kits (Bioclin Laboratories, Belo Horizonte, MG, Brazil), as per manufacturer protocols.

### Enzymatic Activity Assays

Liver homogenates were prepared from 100 mg of tissue for the analysis of antioxidant enzymes, including superoxide dismutase (SOD), catalase (CAT), and glutathione S-transferase (GST). SOD activity was measured using the pyrogallol method,^54^ while CAT activity was evaluated based on the rate of hydrogen peroxide decomposition.^55^ GST activity was determined using the formation of CDNB-glutathione conjugates, as described by Habig et al.^56^ Protein concentration was quantified by the Bradford method.^57^

### Oxidative and Nitrosative Stress Markers

Oxidative damage was evaluated by quantifying malondialdehyde (MDA), a product of lipid peroxidation measured via the TBARS assay as described by Buege and Aust,^58^ and by determining protein carbonyl levels (CP), a marker of protein oxidation, using the DNPH method according to Levine et al.^59^ Nitric oxide production was indirectly assessed by measuring nitrite levels in liver homogenate supernatants, using the Griess reaction.^60^ Total antioxidant capacity was determined using the ferric-reducing antioxidant power (FRAP) assay, which measures the reduction of ferric-tripyridyltriazine to its ferrous form (Fe^2+^), producing a blue color.^61^

### Histopathological and Stereological Liver Analysis

After fixation in Karnovsky’s solution for 24 h, liver fragments were dehydrated in ethanol and embedded in methacrylate (Historesin, Leica Microsystems). Sections (3 μm thick) were stained with hematoxylin and eosin (HE) for histopathology and with periodic acid–Schiff (PAS) for glycogen detection. Digital images were captured using an Olympus BX-53 microscope equipped with an Olympus DP73 camera. All histological and stereological assessments were performed under observer blinding to group allocation. Stereological quantification followed a point-counting approach to estimate the volume density (Vv, %) of liver components—hepatocytes, sinusoidal capillaries, blood vessels, inflammatory infiltrate, and Kupffer cells (macrophages)—according to Vv = PP/PT, where PP is the number of test points hitting the structure of interest and PT is the total number of test points in the sampled histological area.^62^ Analyses were performed on HE and PAS-stained sections at 400× magnification, using a 300-point test grid superimposed on each calibrated field in ImageJ software (National Institutes of Health).

### Granuloma Analysis

Hepatic granulomas were classified into two maturation categories.^40^ Stage 1 (“immature granulomas”) were defined as individual or few fused Kupffer cells with none or few loosely arranged mononuclear cells and a high number of amastigotes; Stage 2 (“mature granulomas”) as tightly fused Kupffer cells surrounded by a more organized cellular infiltrate, with or without collagen deposition, and with some amastigotes still detectable. For each animal, granuloma number was quantified in 10 randomly selected, nonoverlapping fields, and the cross-sectional area of each granuloma was measured on calibrated images in ImageJ (NIH). All assessments were performed under observer blinding to group allocation.

The cross-sectional area of hepatic granulomas was quantified on H&E micrographs using ImageJ (NIH). For each experimental group, ≥ 20 well-delimited granulomas were photographed under identical optics and camera settings. Images were calibrated in ImageJ and each granuloma was delineated manually with the Polygon/Freehand tool following the outer limit of the lymphocytic mantle (peripheral lymphocytic cuff). Area values are reported in square micrometers (μm^2^) after calibration. The adjacent parenchyma and vascular/portal structures were excluded. Confluent or touching granulomas were contoured and recorded individually; transected or poorly delimited lesions were excluded *a priori*. All measurements were performed blinded to group allocation.
